# Molecular Surgery Concept from Bench to Bedside: A Focus on TRPV1+ Pain-Sensing Neurons

**DOI:** 10.3389/fphys.2017.00378

**Published:** 2017-06-02

**Authors:** László Pecze, Béla Viskolcz, Zoltán Oláh

**Affiliations:** ^1^Unit of Anatomy, Department of Medicine, University of FribourgFribourg, Switzerland; ^2^Institute of Chemistry, Faculty of Materials Science and Engineering, University of MiskolcMiskolc, Hungary; ^3^Acheuron Ltd.Szeged, Hungary

**Keywords:** TRPV1, vanilloids, capsaicin, resiniferatoxin, sensory neurons, necrosis, apoptosis

## Abstract

“Molecular neurosurgery” is emerging as a new medical concept, and is the combination of two partners: (i) a molecular neurosurgery agent, and (ii) the cognate receptor whose activation results in the selective elimination of a specific subset of neurons in which this receptor is endogenously expressed. In general, a molecular surgery agent is a selective and potent ligand, and the target is a specific cell type whose elimination is desired through the molecular surgery procedure. These target cells have the highest innate sensitivity to the molecular surgery agent usually due to the highest receptor density being in their plasma membrane. The interaction between the ligand and its receptor evokes an overactivity of the receptor. If the receptor is a ligand-activated non-selective cation channel, the overactivity of receptor leads to excess Ca^2+^ and Na^+^ influx into the cell and finally cell death. One of the best known examples of such an interaction is the effect of ultrapotent vanilloids on TRPV1-expressing pain-sensing neurons. One intrathecal resiniferatoxin (RTX) dose allows for the receptor-mediated removal of TRPV1+ neurons from the peripheral nervous system. The TRPV1 receptor-mediated ion influx induces necrotic processes, but only in pain-sensing neurons, and usually within an hour. Besides that, target-specific apoptotic processes are also induced. Thus, as a nano-surgery scalpel, RTX removes the neurons responsible for generating pain and inflammation from the peripheral nervous system providing an option in clinical management for the treatment of morphine-insensitive pain conditions. In the future, the molecular surgery concept can also be exploited in cancer research for selectively targeting the specific tumor cell.

## The concept of molecular surgery and related technologies

Our goal with this review is to summarize the basics behind “molecular surgery,” a new concept of biomedical technology, which have prototyped with the vanilloid receptor type 1 (TRPV1) target. Currently, resiniferatoxin (RTX) is the number 1 drug candidate to implement the “molecular neurosurgery” technology at cellular levels. To demonstrate the safety and efficacy of the molecular neurosurgery, Dr. Michael J. Iadarola and Dr. Zoltan Olah have initiated a bench-to-bedside (B2B) project in 2000 that has recently entered Phase II (Brown D. C. et al., 2015). Headed by clinicians, the bedside team has already recruited more than 30 cancer pain patients in the clinical trial, which represents a novel and unique treatment option in clinical management of morphine-insensitive cancer pain (Brown D. C. et al., 2015).

The current clinical protocol prescribes a single dose of intrathecal RTX, to treat pain with an agonist of TRPV1 channel. TRPV1 was molecularly identified in 1997 (Caterina et al., 1997). TRPV1 belongs to the diverse transient receptor potential (TRP) family of non-selective cation channels (Benemei et al., 2015). The human TRP superfamily is composed of 27 members which are grouped into six subfamilies based on their amino acid sequence homology: canonical (C), vanilloid (V), melastatin (M), polycystin (P), mucolipin (ML), and ankyrin (A). They all share the common feature of six transmembrane domains and permeability to cations (Montell, 2005).

TRPV1 is dominantly expressed at the source of the inflammatory pain signals (Patapoutian et al., 2009), thus agonist-mediated removal of specific TRPV1+ inflammatory pain-sensing neurons, or “acheurons,” as we call them, can manage even severe chronic pain situations. The detailed mechanism, as it has been revealed, is the RTX-induced cytotoxicity, which exploits the high specificity and affinity of this exovanilloid to TRPV1. This induces a subsequent flux of Ca^2+^ and Na^+^ ions into the acheurons (Olah et al., 2001; Karai et al., 2004; Pecze et al., 2013).

One of the preferred goals of the clinical trial, started in 2008 in the National Institutes of Health (NIH, Bethesda, Maryland), is to manage morphine-insensitive, and subsequently unbearable pain cases that are currently an unmet medical need. The trial also will provide evidence on better end-of-life quality and palliative care of cancer patients and deliver the proof of concept of molecular neurosurgery: only TRPV1+ acheurons, a verified subset of sensory neurons, can be removed by RTX-induced cytotoxicity. The technology is amenable due to the fact that it is supported by a number of experiments carried out in various mammals, from rodents to primates (Olah et al., 2001; Karai et al., 2004; Brown et al., 2005; Tender et al., 2005; Brown D. C. et al., 2015; Brown, 2016). The methods and kits for the selective ablation of pain-sensing neurons have been patented (Iadarola et al., 2012).

Although there are drug leads acting as TRPV1 inhibitors in different stages of R&D pipelines at a number big pharma companies (Kaneko and Szallasi, 2014), currently, there is no vanilloid drug on the market other than capsaicin (CAP). In contrast to RTX, however, CAP is not an optimal compound to target TRPV1 and implement the molecular neurosurgery technology. First of all, ours and others' experiments validated that CAP is less potent as an agonist of TRPV1 than RTX (Szallasi and Blumberg, 1999). In general, one can say that RTX acts in a low nanomolar range on TRPV1, while CAP acts in a low micromolar range, but the exact EC50 values vary between assays (Szallasi and Blumberg, 1999; Olah et al., 2001). As many pharmaceuticals may lose their specificity at higher doses, TRPV1-independent cytotoxic effects have been reported at concentrations above 10 micromolar for RTX and above several hundred micromolar for CAP, tested on Sf9 insect cells that do not have the TRPV1 gene (Pecze et al., 2008).

The vanilloid-binding site of TRPV1 is mapped to a protein region embedded in the lipid membrane, which justifies the use of the more lipophilic RTX. Thus, currently RTX is the vanilloid with the highest affinity and efficacy. We have also determined that CAP, due to its lower affinity and quicker dissociation from the receptor, is an inappropriate drug for implementation of the molecular neurosurgery and unable to deliver robust agonist-induced cytotoxicity within minutes, as noted with RTX, even *in vivo* (Olah et al., 2001; Karai et al., 2004; Brown et al., 2005; Tender et al., 2005).

## Extended use of the agonist-induced cytotoxic mechanism for pain management

TRPV1 channels are highly expressed on C- and Aδ-type sensory neurons. The cell bodies of somatic sensory afferent fibers lie in the dorsal root ganglia (DRG) and trigeminal ganglia (TG). TRPV1 can be stimulated by (i) endovanilloids, produced naturally by peripheral tissues in response to injury, (ii) heat source of moderately high temperature (42–49°C), and (iii) extracellular acidosis (pH ~6.0; Caterina et al., 1997; Tominaga et al., 1998). Endovanilloids are defined as endogenous ligands of TRPV1 (van der Stelt and Di Marzo, 2004). Three different classes of endogenous lipids have been found recently that can activate TRPV1, and these are unsaturated N-acyldopamines, lipoxygenase products of arachidonic acid and linolenic acid, and the endocannabinoid anandamide (van der Stelt and Di Marzo, 2004). These compounds are produced at the site of inflammation. Endogenous TRPV1 ligands have different pharmacological properties (e.g., affinity, potency, metabolic rate, etc.) compared to naturally occurring exogenous agonists such as CAP or RTX, and consequently endogenous ligands have different physiological functions. As an example, endogenous agonists are involved in the generation of chronic pain, while exogenous agonists are capable of alleviating chronic pain (Carnevale and Rohacs, 2016). Potent vanilloids such as CAP or RTX can be administered in a different manner for the removal of TRPV1+ neurons. Routes of administration include (I) topical epicutaneous (application onto the skin), (II) intraarticular, (III) intrathecal (IV) intraganglionic, and (V) systemic intraperitoneal.

- Topical CAP has been used for medicinal puposes for centuries, mainly to treat toothache. Creams containing CAP, generally in the range of 0.025–0.1% by weight, are now available in many countries, and often do not require a prescription, for the management of neuropathic and musculoskeletal pain. CAP creams have shown analgesic benefits in postherapeutic neuralgia, painful polyneuropathies including diabetic and HIV-related neuropathy, and postmastectomy/surgical neuropathic syndromes (Jorge et al., 2010). The CAP 8% patch is approved by FDA (U.S.Food and Drug Administration) for postherapeutic neuralgia. Epidermal nerve fiber density in skin areas exposed to the high-concentration CAP patch (8%) was clearly lower 1 week after a single 60-min application as compared with control biopsies, but at 24 weeks, epidermal nerve fiber density appears similar to the control (Kennedy et al., 2010). Topical RTX administration was studied for treatment of ophthalmic pain. In rat cornea, a single application of RTX dose-dependently eliminated the CAP-induced eye-wiping response for 3–5 days (Bates et al., 2010). This analgesic effect was fully reversible (Bates et al., 2010). The distant surviving neuronal body generates new C-, and Aδ-afferents, which repairs inflammatory pain sensation (Donnerer, 2003; Kennedy et al., 2010). Thus, the analgesic effect of topical vanilloid administration is reversible.
- In the sinovial fluid of joints, RTX can inactivate the C-, and Aδ-nerve pain signaling, but only temporarily.These nerves recover in a month due to fiber regeneration, guided by the unaffected axons in the nerve (Neubert et al., 2003; Kissin E. Y. et al., 2005). This process should be much faster and more complete after intra-articular than sciatic nerve RTX administration, due to the shorter part of the ablated nerve fibers in the first case. These experimented approaches will provide the possibility for a reversible interruption of nerve fiber signaling without their complete and irreversible destruction (Neubert et al., 2003; Kissin E. Y. et al., 2005; Kissin I. et al., 2005).
- In contrast to topical administration, other routes of application generate irreversible changes, because these treatments eliminate not only the periferal axons, but also the body of the TRPV1-positive sensory neurons. Complete removal of the pain sensing neurons are a treatment option for chronic, uncurable pain conditions, such as cancer pain. RTX can be equally potent as an ablative agent of C-fiber and Aδ-afferents in patients with non-terminal stage of diseases coupled with neuropathic complication, including chronic phantom pain and type 2 diebetes (Karai et al., 2004; Brown et al., 2005; Mannes et al., 2005; Neubert et al., 2005; Tender et al., 2005). We and others have collected the necessary evidence for the extended use of the agonist-induced cytotoxic mechanism to carry out molecular neurosurgery. Due to the extreme specificity of RTX to TRPV1, the cytotoxicity does not affect other sensory modalities, such as super-heat, cold, light touch, noxious mechano-, and proprioceptive sensations. The RTX-assisted neurosurgery neither impairs bystander motor neurons nor influences consciousness (Olah et al., 2001; Karai et al., 2004; Brown et al., 2005; Tender et al., 2005; Patapoutian et al., 2009). The safety and efficacy of RTX have successfully been validated even in dogs and monkeys (Olah et al., 2001; Karai et al., 2004; Brown et al., 2005; Tender et al., 2005; Gunthorpe and Szallasi, 2008).
- In contrast to intrathecal administration, intraganglionic injection may need an advanced imaging technology R&D process, in order to fully support robotic anatomical guidance (i.e., needle placement by computer tomography). With this technology at hand, we can treat severe focal pain syndromes right at the source, and remove acheurons from a sub-domain of DRG or TG branches. One of the well-defined medical needs in trigeminal neuralgia requires CT-guided needle placement to inject RTX with an intra-nerve anatomic precision (Brown J. D. et al., 2015).
- Prior to transgenic TRPV1 knockout models, systemic chemo-denervation was employed with potent vanilloids (Jancso et al., 1967; Szolcsanyi et al., 1990) to study animal behavior with altered pain sensations. Respiratory depression represents the limiting factor in rats for acute and systemic administration of CAP or RTX (Szallasi et al., 1989). The therapeutic window for RTX is wider than for CAP. RTX administered at a dosage of 50 μg/kg body weight effectively removed all pain-sensing neurons in young adult mice (Pecze et al., 2009). Mice survived the procedure and lived to an old age in the animal facility. They remained fertile, however they were not able to adapt to heat stress (Pecze et al., 2009). If subjected to elevated ambient temperature (38°C), RTX-treated rats showed a steady rise in body temperature, ultimately leading to collapse, in contrast to control animals, which did not show these behaviors (Szallasi and Blumberg, 1989).

In conclusion, RTX usually provides a better pharmacological profile than CAP. CAP is effective in micromolar concentrations and ranked approximately three factors of magnitude less potent as a vanilloid agonist than RTX. Although RTX is more potent, paradoxically it evokes less pain feeling than CAP, because the initial activation of the pain pathway *via* TRPV1 is immediately cut by the ionic influx-induced fragmentations of pain-sensing C- and Aδ-fibers. Even an intrathecally administered 1 μg/kg dose of RTX can rapidly eradicate the inflammatory pain signaling by a robust TRPV1-amplified cytotoxicity without significant side effect, tested in patient dogs suffering either cancer or osteoporosis pain (Brown et al., 2005). At the site of application the action potential conductivity of the acheuron's membranes is blocked in seconds, either when RTX is given proximally (i.e., intrathecal, intraganglionic) or distally (i.e., transdermal) to the neuronal body. Thus, only a small dosage of noxious stimuli can reach the central nervous system (Caudle et al., 2003; Neubert et al., 2003; Karai et al., 2004). In the veterinary practice, RTX-treatments of severe cancer and osteoporotic patient dogs have demonstrated initial, short-lived physiological changes, but then the blood pressure and cardiac parameters went back to the normal range. The benefit of longterm and permanent elimination of unbearable inflammatory pain justifies the effective ablation of acheurons (Karai et al., 2004; Tender et al., 2005).

## Empirical usage of molecular surgery

Nowadays, rational and mechanism-based applications of technologies based on specific molecular surgery agents can replace previous empiric practices. The surface of the human body, externally, while the gastrointestinal system, internally, are continuously exposed to pungent compounds such as CAP, gingerol, piperine, allyl-isothiocyanate (i.e., chili pepper, ginger, black pepper, and mustard, respectively), and other phytochemicals from spices for hundreds or thousands of years (Iwasaki et al., 2008; Masamoto et al., 2009). Likewise, either by chance or conscious use, RTX had been applied in human since the ancient times. Tribal witchdoctors in Africa often administered the latex from different Euphorbia species to the wound after removal of a tooth to diminished pain and inflammation. Empirically, a number of pungent vanilloids supplement our daily meals. Various Hungarian dishes are made exclusively with hot peppers, so the human body is frequently exposed to vanilloids. In Asia as well, different pungent bioactive ingredients are used in their daily diet and target either the TRPV1 or TRPA1 channels. Thus, many of the agents mentioned in this review are administered to humans, either by frequent consumption or empiric application, first as folk medicine, then in medical practice (Sterner and Szallasi, 1999; Szallasi and Blumberg, 1999). Epidemiologic evaluations and natural uses have demonstrated that these bioactive phytochemicals can save medical expenses and prolong lifespan. A large cohort study revealed that consumption of spicy food is inversely correlated with the mortality caused by cancer, ischemic heart diseases and respiratory diseases (Lv et al., 2015).

## The role of advanced imaging tools

**Figure 1:**
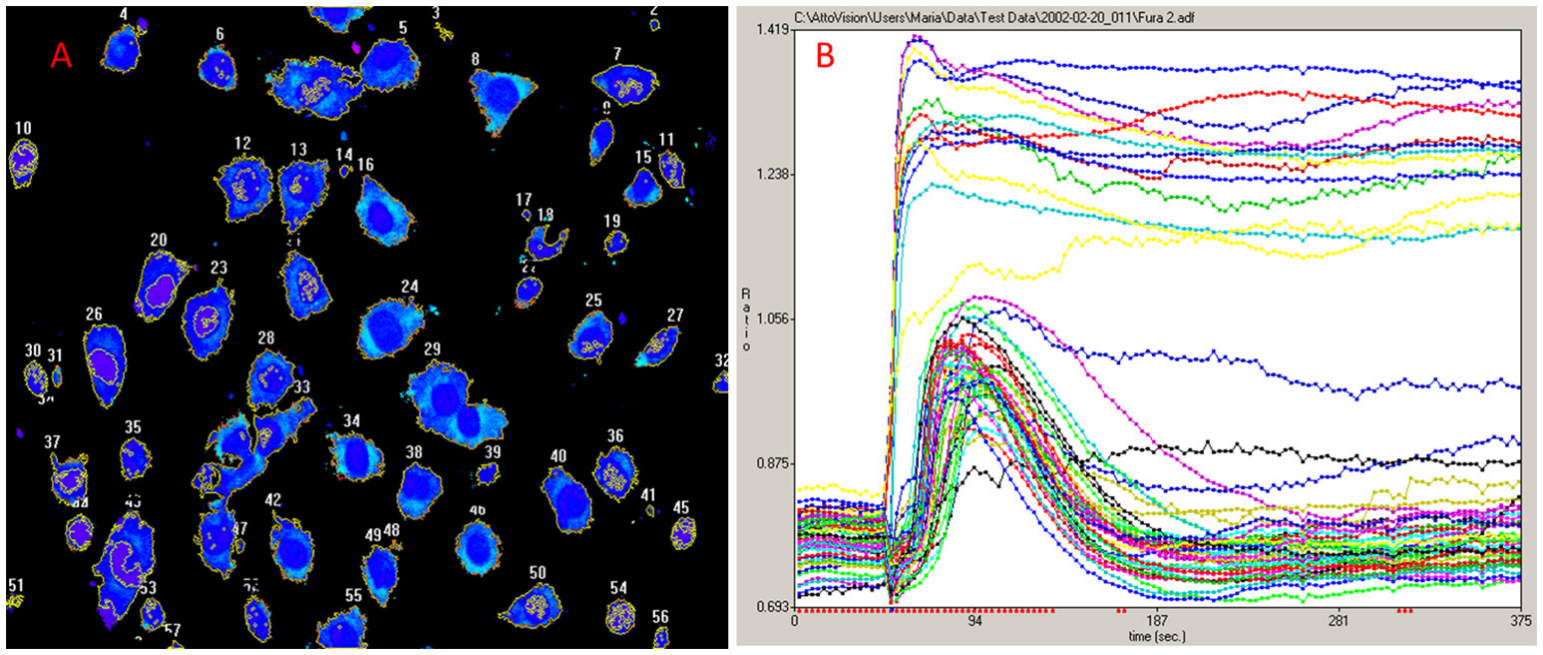
Ca^2+^ imaging. **(A)** NIH-3T3 cells ectopically expressing TRPV1 were loaded with the cytosolic Ca^2+^ indicator Fluo-4-AM. **(B)** Minute scales imaging of [Ca^2+^]_i_ reveals two populations in NIH-3T3^TRPV1^ cell line. A set of cells responds to 1 μM CAP with non-declining increase of [Ca^2+^]_i_ and dies very soon while the other population survives the initial necrotic-phase in which the [Ca^2+^]_i_ transitions back to closed to its resting levels. This later option rarely occurs with RTX, as RTX is several thousandfold more potent than CAP in several assays (Szallasi and Blumberg, 1999). The original experiment was published in 2004 (Karai et al., 2004).

**Figure 2:**
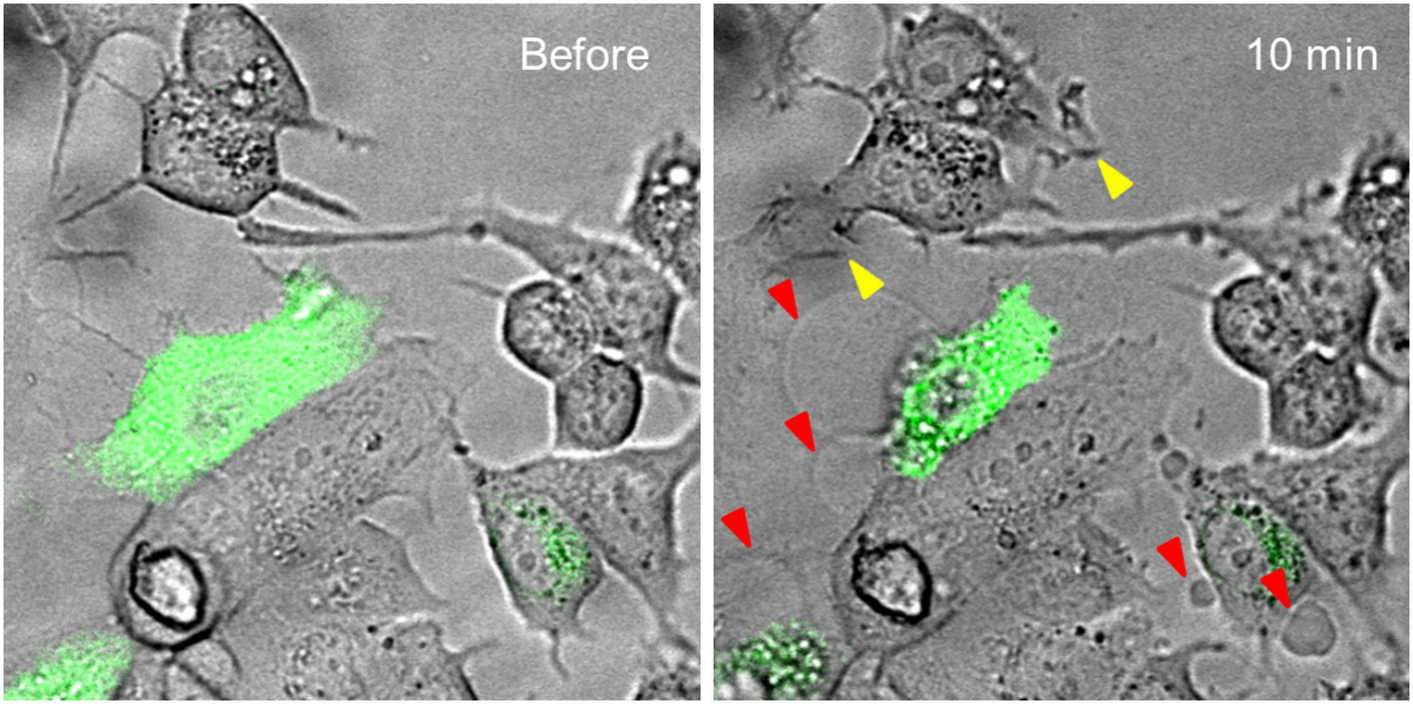
Time-lapse analysis of vanilloid evoked structural changes in MCF7 breast cancer cell line. MCF7 cells were transiently transfected with TRPV1-GFP construct. These show green fluorescence. Cells were treated with 50 μM CAP. TRPV1-GFP expressing cells, and only these cells, blow blebs during a 10-min period (red arrowheads). MCF7 cells also endogenously express low levels of TRPV1 channels. This level is not enough to induce bleb formation, but some MCF7 cells produce invadopodium, an ameboid structure promoting cancer cell invasion, in response to CAP treatment (yellow arrowheads). The original experiment was published in 2016 (Pecze et al., 2016b).

For the exploration of the function of the TRPV1 channel, confocal fluorescence microscopy was vastly instrumental. Firstly, confocal microscopy was used for the imaging of dynamics in intracellular free Ca^2+^ concentrations ([Ca^2+^]_i_) (Figure 1). Moreover, by tagging of TRPV1 channel with fluorescent proteins, the mechanism of cell death became visually trackable in real-time (Figure 2 and Supplementary Video; Olah et al., 2001). Both N- and C-terminally tagged TRPV1 proteins results in fully functional TRPV1 channels. Cells ectopically expressing the TRPV1 receptor show necrotic bleb formation upon CAP stimulation (Pecze et al., 2013). Bleb formation is dependent on both Ca^2+^ and Na^+^ influx (Pecze et al., 2016b). Bleb formation can be so intensive that the cell blows up, until finally the loss of the plasma membrane integrity leads to necrotic cell death (Pecze et al., 2016b). Besides this, cell organelles such as the mitochondria and endoplasmic reticulum also show fragmentization within 1 h (Olah et al., 2001). High resolution confocal images has helped in the figuring out of the molecular neurosurgery mechanism and in distinguishing the difference between the efficacy of CAP and RTX (Olah et al., 2001). Cells ectopically expressing fluorescently tagged TRPV1 channels were voltage-clamped and 10 μM CAP induced large inward currents similar to that of 125 pM RTX. Capsazepine, a competitive TRPV1 antagonist, attenuated the vanilloid-induced currents (Olah et al., 2001; Liu et al., 2003; Marshall et al., 2003).

Fusion of TRPV1 with rationally chosen fluorescent protein allows for co-localization studies which exploit the fluorescence resonance energy transfer (FRET) phenomenon (Hellwig et al., 2005; Zagotta et al., 2016). The optical sectioning capabilities of confocal fluorescence microscopes followed by 3D reconstruction revealed the innervation pattern of the epithelium of guinea-pig trachea by TRPV1 immunoreactive axons (Watanabe et al., 2005). Confocal images showed the loss of TRPV1-immunoreactive DRG neurons and afferent terminals in the spinal cord after RTX treatment (Chen and Pan, 2006).

Ectopically expressed fusion proteins allowed for the determination of the sub-cellular distribution of TRPV1 receptors. It became clear that in addition to the plasma membrane (PM), where previously, TRPV1 was expected to mechanistically localize (TRPV1_PM_), it was also found and noted to operate in the endoplasmic reticulum (TRPV1_ER_), as well (Olah et al., 2001; Karai et al., 2004). The endoplasmic reticulum (ER) is the major intracellular storage of Ca^2+^ ions and like TRPV1_PM_, the TRPV1_ER_ receptor is also gated by vanilloids and contributes to the agonist-induced cytotoxicity. The potency of less lipophilic CAP on TRPV1_ER_, is likely hampered by its slow membrane penetration and distribution to deeper cellular compartments (Lazar et al., 2006).

## Mechanism of action

In physiological conditions, TRPV1 is activated by the endogeneous ligand produced at the site of inflammation or tissue injury. TRPV1 is a non-selective cation channel with a higher permeability for divalent cations, such as Ca^2+^ (permeability ratio P_Ca_/P_Na_ is around ~10; Gees et al., 2010). However, both Ca^2+^ and Na^+^ influxes through the TRPV1 channel play a role in the transmission of nociceptive signals from the periphery toward the central nervous system. Besides this, activation of TRPV1 causes cell depolarization. Sensory neurons as excitable cells express voltage-operated ion channels. Activation of TRPV1 channels triggers the gating of those channels. The firing pattern of neuronal cells is modulated by conductance changes *via* TRP channel activation or inhibition (Gees et al., 2010). It is worth noting that TRPV1 produces an analog Ca^2+^ signal i.e., the amount of Ca^2+^ ions passing through the channel is proportional to the stimulus intensity. The activated inositol phospholipid pathway acts as an amplifier and frequency-based modulator on Ca^2+^ signals produced by TRPV1. The frequency of the intracellular Ca^2+^ oscillations are related to the strength of TRPV1 stimulation (Pecze et al., 2016a). Since exogenous TRPV1 ligands (CAP and RTX) have different pharmacological properties such as higher affinity and potency compared to endogenous agonists, they consequently induce an over-activity of the TRPV1 receptor. Thus, RTX and CAP induce a prolonged increase in [Ca^2+^]_i_, but only in sensory neurons expressing TRPV1 while not in other cells (Olah et al., 2001; Karai et al., 2004).

## Desensitization vs. deletion of a cell/neuron

Desensitization is the phenomenon in which a receptor's responsiveness decreases after continued or repeated stimulation with an agonist. Prolonged or repeated applications of CAP causes persistent desensitization of TRPV1 in an electrophysiology-based experiment (Touska et al., 2011). Although this effect can also contribute to pain insensitivity after vanilloid treatment to some extent, our experiments show that sensory neurons or axons were absent in the treated region. Thus, in contrast to desensitization, an alternative mechanism of potent vanilloids has been proposed; complete removal of TRPV1-specific nociceptive neurons is the cause of the long-lasting/permanent inflammatory pain-free state (Olah et al., 2001; Caudle et al., 2003; Karai et al., 2004) and these findings were later confirmed by others (Chen and Pan, 2006; Kennedy et al., 2010; Kun et al., 2012).

However, controversy regarding nerve fiber degeneration vs. long-lasting desensitization without neuronal degeneration still exists in terms of the explanation for the mechanism of potent vanilloid agonism. Prior to transgenic TRPV1 knockout models, chemo-denervation was employed with potent vanilloids (Jancso et al., 1967; Szolcsanyi et al., 1990) to study animal behavior without pain sensation. Although the inflammatory pain-free state that either CAP or RTX treatment produced was unusually long lasting (Szallasi et al., 1989; Szallasi and Blumberg, 1992), more than minutes or hours, the early explanations of the analgesic actions of vanilloids suggested a desensitization of nerve terminals (Szolcsanyi et al., 1975). Thus, the literature still uses long-lasting desensitization as an explanation.

## Necrotic vs. apoptotic processes

It was determined in a number of experiments that RTX (i.e., 1 μg/kg) produces analgesia by robust Ca^2+^-mediated cytotoxicity, if applied (i) intradermally, lasting for several days to a month, or (ii) intrathecally and intraganglionically, permanent for a lifetime, removing the entire TRPV1+ neuron (Szabo et al., 1999; Karai et al., 2004).

The cellular and molecular mechanisms underlying the vanilloid-induced neural loss are still unresolved. Evidence for CAP-induced neuronal cell death by apoptosis with caspase activation has been reported (Shin et al., 2003; Jin et al., 2005), while some studies state that it is an apoptosis-like, but caspase-independent process (Movsesyan et al., 2004; Davies et al., 2010). Still other studies doubt the process to be apoptotic at all in nature (Olah et al., 2001; Caudle et al., 2003). It is very likely that both apoptosis and necrosis might play a role in TRPV1-mediated toxicity, depending on the strength of the activation and moreover on the experimental protocol. The Ca^2+^ ionophore ionomycin acts by creating Ca^2+^-permeable pores in cell membranes. Analagous to TRPV1-related cytotoxicity, it can induce either apoptosis or necrosis in cultured cortical neurons (Gwag et al., 1999).

**Figure 3:**
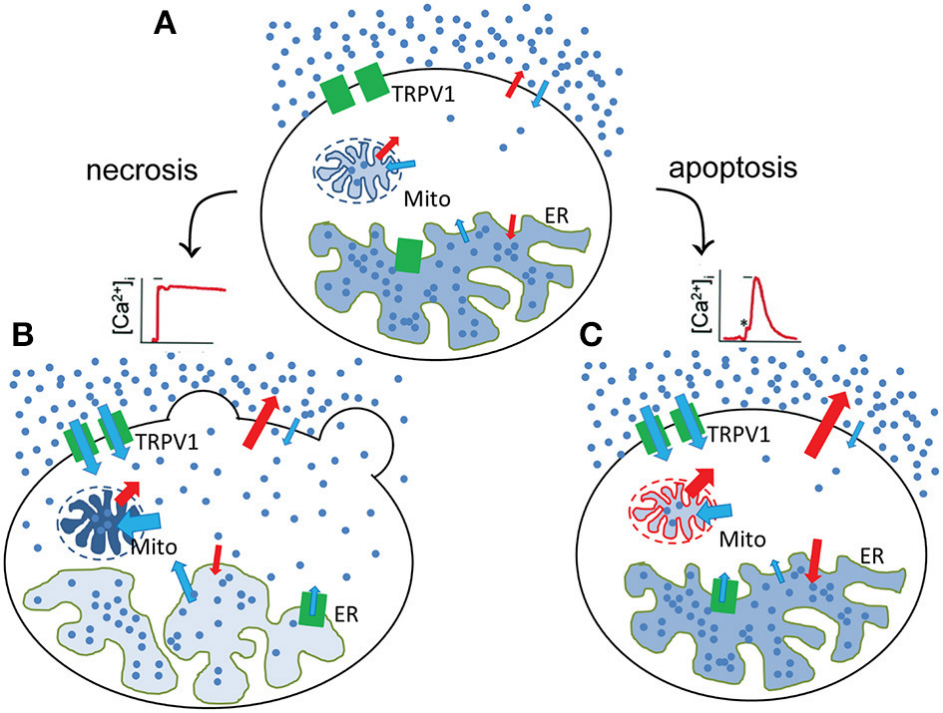
Necrotic and apoptotic processes after TRPV1 stimulation. **(A)** In the unstimulated state, the resting [Ca^2+^]_i_ is the result of the low rate of influx and efflux across the plasma, ER- and mitochondria membranes. Red and blue arrows indicates the energy-requiring and energy-independent fluxes, respectively. After stimulation, two types of Ca^2+^ response can be observable leading to necrotic **(B)** and potentially apoptotic processes **(C)**. **(B)** During the necrotic processes TRPV1 activation results in a sustained increase in [Ca^2+^]_i_. After that, Ca^2+^ ions are accumulated in the mitochondria (Pecze et al., 2016b) but released from the endoplasmatic reticulum. These processes lead to the fragmentation of these organelles (Olah et al., 2001). Blebs appears at the plasmamembrane due to the cell volume increase. **(C)** During the apoptotic processes TRPV1 activation does not result in a sustained increase in [Ca^2+^]_i_, but rather to a transient Ca^2+^ signal mainly due to the depletion of the ER Ca^2+^ stores. In this situation, Ca^2+^ extruding systems is still able to create an equilibrium between the Ca^2+^ influx and Ca^2+^ efflux reverting [Ca^2+^]_i_ close to its basal levels before stimulation. However, this new equilibrium requires elevated energy consumption. Mitochondria therefore produce more energy, but during their normal operation they also produce reactive oxygene species (ROS; Michael Murphy, 2009). ROS production was significantly increased in cultured DRG neurons after bath application of CAP (1 μM) or RTX (200 nM) compared with the untreated neurons (Ma et al., 2009). This can induce oxidative stress and apoptosis Fleury et al., 2002.

The MCF7 breast cancer cell line, although it expresses endogeneously low levels of TRPV1 receptors, cannot be subjected to necrotic-like procecesses by administration of CAP or RTX, but shows the typical structural changes when TRPV1 is ectopically overexpressed (Figure 2). Interestingly, in MCF7 cells, the mere overexpression of GFP-tagged TRPV1 channels decreased cell viability (Pecze et al., 2016b). We observed that mainly apoptotic processes were activated, but mitotic arrest in MCF7^GFP-TRPV1^ cells was also detected. The absence of mitosis in the surviving MCF7^GFP-TRPV1^ cells subsequently did not allow for the establishment of stable MCF7^GFP-TRPV1^ clones, although we had been successful in establishing cell clones permanently expressing ectopic TRPV1 proteins using non-tumor-derived cell lines such as HaCaT, a spontaneously immortalized keratinocyte cell line from adult human skin (Pecze et al., 2008), or NIH-3T3 cells, a spontaneously immortalized mouse embryo fibroblast cell line (Olah et al., 2007). Moreover, prolonged treatment of non-transfected MCF7 cells with CAP induces apoptotic processes due to increased oxidative stress (Kosar et al., 2016). The supposed mechanisms of necrotic and apoptoic processes are summarized in Figure 3.

## Safety and efficacy of molecular surgery agents in humans

Resistance to vanilloids provides additional safety and efficacy to the molecular surgery technology. We and others have noted that TRPV1 and its mRNA are detected in a broader spectra of cells rather than only from DRG or TG origins. Paradoxically, the occurrence of TRPV1 does not necessarily mean that the cell can automatically be deleted by the vanilloid-mediated molecular surgery. Vanilloid binding cannot be mechanistically linked either to channel opening or to permanent elevation in [Ca^2+^]_i_. This issue has been addressed in studies of human keratinocytes (Pecze et al., 2008; Kun et al., 2012).

One potential explanation for vanilloid resistance is that TRPV1 subunits need to form a homotetramer channel, a quaternary structure required for maximal vanilloid sensitivity and channel activity (Kedei et al., 2001; Garcia-Sanz et al., 2004; Moiseenkova-Bell et al., 2008). Any obstacles that hamper quaternary structure formation of the pore from the subunits may reduce the cell's sensitivity to vanilloids. Mounting evidence shows that TRPV1 is capable of heteromerization with other TRP channel homologs upon co-expression (Fischer et al., 2014). Major determinants of TRPV1 oligomerization have recently been localized to the C-terminal ^684^Glu-^721^Arg, the so-called association domain (Garcia-Sanz et al., 2004). Recombinant association domains form stable multimers, however, association domain-deleted TRPV1 is monomeric and blocks self-assembly of wild-type subunits in functional homotetramers. Evolutionarily conserved, homologous, but not identical, association domains, however, may allow for the combinatorial assembly of different TRP channels that are gated by distinct ligands other than vanilloids.

Besides this, our experiments indicate that TRPV1 channel density on the plasma membrane is an important factor for the cell's sensitivity to vanilloids (Pecze et al., 2016b). Cells expressing lower density of the TRPV1 channel are evidently more resistant to TRPV1-mediated cytotoxicity. Unfortuntely, although tumor cells express higher levels of TRPV1 than normal epithelial cells, they still do not have enough receptors to perform tumor-targeted TRPV1-mediated necrotic-type eradication (Pecze et al., 2016b). Nevertheless, several experiments (Diaz-Laviada and Rodriguez-Henche, 2014) as well as a case report (Jankovic et al., 2010) suggest that vanilloids have anti-cancer activity. The origin of the anticancer effects of vanilloids is not completely solved and it needs further examination. Whether it is TRPV1-mediated or a TRPV1-independent effect is still in question (Diaz-Laviada and Rodriguez-Henche, 2014). To make things more complicated, pain sensing neurons innervate the tumor mass and communicate with the tumors (Li et al., 2013). Systemic removal of TRPV1+ neurons in mice increased the number of metastasis of breast cancers (Erin et al., 2004).

## Proof of the efficacy of molecular neurosurgery for the treatment of type II diabetes and urinary disfunctions

The proof of the applicability of molecular surgery reveals a second use of RTX in type II diabetes as an anti-neuropathic treatment agent (Gram et al., 2005; Moesgaard et al., 2005). The system that regulates insulin secretion from beta-cells in the islet of Langerhans has a vanilloid-sensitive inhibitory component. Calcitonin Gene Related Peptid (CGRP)-expressing TRPV1+ primary sensory fibers innervate the islets. The CGRP-containing primary sensory neurons are targets of the RTX-mediated molecular neurosurgery. Elimination of vanilloid-sensitive primary afferents by vanilloids before the development of hyperglycemia prevents the increase of plasma glucose levels and coincides with enhanced insulin secretion and a loss of CGRP-expressing islet-innervating fibers. These data indicate that CGRP-containing fibers in the islets are sensitive to molecular neurosurgery, and that elimination of these fibers contributes to the prevention of the deterioration of glucose homeostasis through increased insulin secretion in genetically obese rats (Gram et al., 2005, 2007; Moesgaard et al., 2005).

Vanilloid-sensitive C- and Aδ-afferents are present in the human bladder's urothelium and are involved in the micturition reflex. Although, intravesically administered RTX most likely acts analogous to CAP, its better pharmacodynamic profile allows for an increase in bladder volume and a higher threshold for the micturition reflex (Payne et al., 2005; Raisinghani et al., 2005). This improvement coincides with a disappearance of CGRP and Substance P (SP) immunoreactive fibers, selective biomarkers of afferents of TRPV1+ neurons. Thus, the loss of CGRP and SP peptide immunoreactivity, consistent either with agonist-mediated depletion of neurotransmitters, or deletion of these fibers by vanilloid-mediated Ca^2+^-cytotoxicity via molecular neurosurgery.

**Figure 4:**
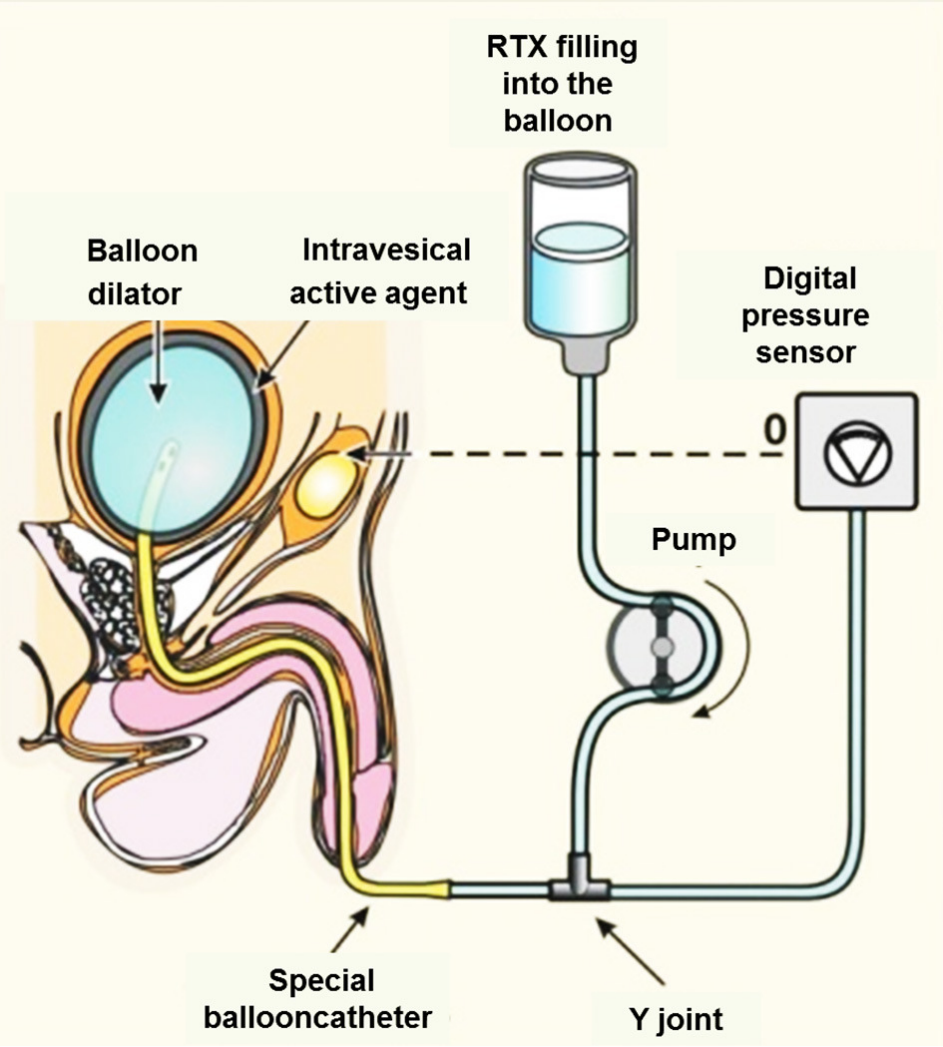
Topical intravesical medication of RTX *via* transurethral instillation promises several advantages over oral systemic CAP therapy. Intravesically administered RTX penetrates the vesical mucosa and submucosa by diffusion and binds to TRPV1+ nerve endings. The suggested “balloon dilator” method benefits from the increased surface of urothelium due to the thinning of the bladder mucosa.

Overactive bladder syndrome, a common type of micturition disorder, can lead to the loss of bladder control, which is then known as urge incontinence. First CAP (Szallasi et al., 1993; De Ridder et al., 1997), then RTX (Lazzeri et al., 1997), were tried as experimental drugs to inactivate incontinency in the clinical settings (Figure 4). It has been long known that these reflexes in the bladder are mediated by C- and Aδ-fiber afferents of nociceptive neurons located in the sacral DRGs. The Afferon Inc., in the late 90's, patented a method of treating neurogenic urinary dysfunction with RTX (Cruz and Agersborg, 2014), and has enrolled patients affected with urge incontinence due to various neurological diseases. The Afferon was admitted into phase II clinical trials. Currently, Eli Lilly and Company has exclusive worldwide license rights for the commercial use of RTX for the treatment of bladder disease or function. Unfortunately, in contrast to these practices (Guo et al., 2013; Foster and Lake, 2014), RTX is still not a registered drug.

Nevertheless, the effectiveness of intravesical RTX treatment strongly varies from study to study. The reasons for these inconsistencies in the clinical outcome might be manifold: too dilute samples of vanilloids were used, different origins of the latent disease resulting in urinary disfunction, or the simple lack of a significant effect of RTX in a specific type of urinary problem. We would like to pay attention to the fact that strong adsorption of RTX and CAP molecules into the tubes of the application devices might also occur. These technical problems can cause a huge variance in the clinical outcomes. Animal experiments will be required to obtain the appropriate material for these tubes. By using a water-soluble formulation of vanilloids (Appendino et al., 2010) the adsorption of vanilloid molecules onto the tubes might also be avoidable. The loss of the TRPV1+ nerve endings in the urothelium can serve as a marker for the successful intravesical instillation.

## Other agonist-activated Ca^2+^-channels as targets of novel molecular surgery agents

The molecular mechanism of TRPV1-mediated cytotoxicity shows conspicuous similarity to glutamate-receptor mediated excitotoxicity, i.e., robust Na^+^ and Ca^2+^ influx, cell swelling, mitochondrial Ca^2+^ loading, and production of reactive oxygen species (Dong et al., 2016). Ionotropic glutamate receptors, as glutamate is the main excitatory neurotransmitters in the central nervous system, play important role in production of excitatory postsynaptic potentials, neuronal migration, synapse formation, learning, and memory (Choi and Rothman, 1990; Dugan et al., 1995). Kainic acid, an agonist for kainate-class ionotropic glutamate receptors, is commonly injected into laboratory animal models to study the effects of experimental ablation. However, attempts to limit cell loss to specific hippocampal neurons have been met with mixed successes and failures (Jarrard, 2002).

The concept of moleculary surgery would be an ideal approach for cancer treatment. Tumors express a different composition of TRP channels than normal cells (Park et al., 2016). Finding a specific TRP target overexpressed only in the tumor cells and finding a potent agonist would provide an ideal pair for the tumor-specific eradication. Recently, it was found that kidney cancer cells can be efficiently and specifically targeted by (-)-Englerin A, a potent and selective activator of TRPC4 and TRPC5 channels (Akbulut et al., 2015). The renal cancer cell line A498, which is most sensitive for (-)-Englerin A, has a highest degree of expression of TRPC4 among the NCI60 cell lines (Akbulut et al., 2015). New derivatives of (-)-Englerin A have been synthetized in order to find effective drugs for the treatment of renal cell carcinoma. A patent application has been filed for this treatment (Echavarren et al., 2011). These new derivatives open a new way of the final goal of finding effective drugs for the treatment of renal cell carcinoma.

## Conclusions and perspectives

Evidence-based and clinically-tried B2B-approaches, such as molecular neurosurgery prototyped with RTX and TRPV1 channel, can be extended for other applications. Here we put an emphasis to the analgesic use of the agonist-induced selective cytotoxic mechanism, however by analogy, a number of robust, biological cell deletion mechanisms may be used in the near future. For example, treatment of metastatic cancers might be amenable by using cancer-specific TRP targets.

Currently, RTX is the most powerful molecular surgery agent in Phase II clinical trials to manage cancer pain in humans. RTX has also been evaluated in severe inflammatory pain states and various neuropathies, as it only removes acheurons and preserves any other bystander cells, fibers, and nerve endings, with little or no side-effects. RTX works in conjunction with TRPV1, with a lack of any effect to cells which do not express TRPV1.

Combination treatment, however, may extend the utility of CAP or other weaker agonists enhancing the cytotoxic effect of vanilloids. A family of positive allosteric modulators (PAM) of TRPV1 only activates Na^+^ and Ca^2+^ entry *via* the vanilloid receptor channel if a vanilloid (CAP, RTX, piperine, etc.) is already bound to the receptor (Roh et al., 2008). These compounds extend the molecular tools of molecular surgery. PAM further increases by 2–3-fold the maximal effect of vanilloids on the induction of Na^+^/Ca^2+^-uptake, producing little or no action when used alone (Roh et al., 2008; Kaszas et al., 2011; Lebovitz et al., 2012). Thus, weaker exovanilloids such as CAP and piperine, present in hot peppers and black peppers, or even weaker endovanilloids can serve as agents to fight against inflammation, pain, and neuropathies. Morevover, RTX, together with a PAM molecule, MRS1477, can provide innovative solutions to number of currently unmet medical needs. The exact mechanism of positive allosteric modulation and the domain specificity of the binding site is not enterely known. Therefore, a more detailed quantitative structure-activity relationship and the determination of the TRPV1-mediated cytotoxic capacity of PAMs must be examined by further studies.

## Author contributions

LP, ZO wrote the review BV critically revised the work and approved its version to be submitted.

### Conflict of interest statement

ZO is named as an inventor, but not the owner of a patent #US8338457 B2 related to this study. Otherwise, the authors declare that the research was conducted in the absence of any commercial or financial relationships that could be construed as a potential conflict of interest.

## Abbreviations

RTX: resiniferatoxin
CAP: capsaicin
TG: trigeminal ganglia
DRG: dorsal root ganglia
FDA: Food and Drug Administration
*NIH*: National Institutes of Health
PAM: positive allosteric modulator
CGRP: Calcitonin Gene Related Peptide
SP: Subtance P
B2B: bench-to-bedside.
